# Honey-based hydrogel: *In vitro* and comparative *In vivo* evaluation for burn wound healing

**DOI:** 10.1038/s41598-017-08771-8

**Published:** 2017-08-29

**Authors:** Reham F. El-Kased, Reham I. Amer, Dalia Attia, M. M. Elmazar

**Affiliations:** 1 Department of Microbiology & Immunology, Faculty of Pharmacy, The British University in Egypt, BUE Cairo, Egypt; 2 Department of Pharmaceutics and Industrial Pharmacy Faculty of Pharmacy, October, University for Modern Sciences and Arts (MSA), Cairo, Egypt; 3 Department of Pharmaceutics, Faculty of Pharmacy, Al-Azhar University, Cairo, Egypt; 4 Department of Pharmaceutics, Faculty of Pharmacy, The British University in Egypt, BUE Cairo, Egypt; 5 Department of Pharmacology, Faculty of Pharmacy, The British University in Egypt, BUE Cairo, Egypt

## Abstract

Honey was used to treat wounds since ancient times till nowadays. The present study aimed at preparing a honey-based hydrogel and assay its antimicrobial properties and wound healing activity; *in-vitro* and *in-vivo*. Topical honey hydrogel formulations were prepared using three honey concentrations with gelling agents; chitosan and carbopol 934. The prepared formulae were evaluated for pH, spreadability, swelling index*, in-vitro* release and antimicrobial activity. The pH and spreadability were in the range of 4.3–6.8 and 5.7–8.6 cm, respectively. Chitosan-based hydrogel showed higher *in-vitro* honey release with diffusional exponent ‘n ≤ 0.5 indicates Fickian diffusion mechanism. Hydrogel formulae were assessed for *in-vitro* antimicrobial activity using Disc Diffusion antibiotic sensitivity test against common burn infections bacteria; *Pseudomonas aeruginosa*, *Staphylococcus aureus*, *Klebsiella pneumonia* and *Streptococcus pyogenes*. The 75% honey-chitosan hydrogel showed highest antimicrobial activity. This formula was tested for *in-vivo* burn healing using burn-induced wounds in mice. The formula was evaluated for burn healing and antibacterial activities compared to commercial product. 75% honey-chitosan hydrogel was found to possess highest healing rate of burns. The present study concludes that 75% honey-chitosan hydrogel possesses greater wound healing activity compared to commercial preparation and could be safely used as an effective natural topical wound healing treatment.

## Introduction

Burns are a common health problem in both developing and industrialized countries. Burns in developing countries, however, may result in serious complications due to the low public hygiene measures which might lead to secondary bacterial infections with need to urgent use of antimicrobial agents. However, the wide use of antibiotics has led to a rise in microbial drug resistance, resulting in poor treatment efficacy and significant economic loss^1^.

A variety of commercially available wound dressings were launched in recent years, yet they possess certain critical limitations such as addition of antimicrobial agents, which might possess cytotoxic effects, especially on prolonged treatment period, leading to delayed wound healing. A large number of the marketed dressings lose their moisturizing effect which makes them adhere to the surface of the wound and damage the newly formed epithelium. The need for effective natural products, such as honey, is essential to overcome the problems of using chemotherapeutics.

Honey is a nutritious thick carbohydrate-rich syrup, which was effectively used since ancient times in traditional medicine. Nowadays, honey is widely used due to its evidenced broad therapeutic uses. It is a well-known antibacterial^2^, anti-parasitic^3^, pain-reliever^4^ and it has proven efficiency against respiratory tract infections^5^. Honey has the ability to stimulate human monocytes to produce cytokine. Recently, honey showed antineoplastic activity using an induced bladder cancer model^6^.

Using honey in treating burns has the advantage of creating a moist environment, it saves the integrity of the burn surface as it is non- adherent, it provides a bacterial barrier that prevents cross-infection and prevents infecting bacteria. Antibacterial property of honey is attributed to its high osmolarity, low pH and hydrogen peroxide production^7^. Many reports address the antibacterial properties of honey against a wide variety of microbes which accelerates wound healing process^7–9^. The phytochemical components of honey; flavonoids and phenolic acids, play a major role as antioxidants due to their free radical scavenging activities which guards cells from the damage due to free radicals and eventually decrease the inflammatory response^8, 9^. Honey has anti-inflammatory properties as it debrides the wound, inhibits scarring and it also encourages wound healing by stimulating tissue regeneration in addition to reducing the need for skin grafting. There are no adverse effects from using honey in burn healing^10^.

The physical properties of honey makes it difficult for direct application on an affected area, as it may liquefy at skin burn temperature as it becomes more fluid at higher temperatures; this restricts the body location on which it can be used, due to the problem of liquefaction and leakage also leading to difficulties in maintaining the required therapeutic concentration for enough time.

The previous limitations can be solved by incorporating honey in a hydrogel formula. Literatures have reported that hydrogel technology deliver products appropriate for medical use particularly in wound management^11–13^. Providing a moist environment and good fluid absorbance which are needed for a successful wound healing process are generously provided by hydrogels^11^. Hydrogel pharmaceutical preparation is preferred over creams because of the high water content in gels which help in pain reduction at the time of application, particularly to mucous membranes and in injured or burned skin^14^. Hydrogels have several favorable properties over ointments when intended for dermatological use such as being emollient, thixotropic and greaseless. Hydrogels also spread more easily and are easily removable, non-staining, compatible with several excipients and water-soluble or miscible^15^. Therefore, developing hydrogel-based wound dressings became of major interest using a wide variety of biocompatible matrices and biologically active substances such as chitin, chitosan, alginate and the neutralized polyacrylic acid (PAA) (carbopol)^16–19^. Chitosan, a widely used natural polymer, is obtained from the skeletal materials of crustaceans, cuticles of insects, and cell walls of many fungi. It is a polysaccharide comprising copolymers of glucosamine and N acetylglucosamine. Chitosan can be prepared by N-deacetylation of chitin^20, 21^. Chitosan is biodegradable^22^ biocompatible^23, 24^, non-toxic and have bio-adhesive features^25, 26^ making it a great choice for integration in topical burn healing pharmaceutical dosage preparations. Chitosan enhances the epithelisation process^27^ and thus possess an accelerating effect on wound healing progress. Scientists are currently in the process of synthesizing the chitosan based hemostatic dressing as it has a promising outcome on decreasing pre-operative and post-operative bleeding^28^. In the utilization of chitosan material, hydrogels is a major branch, since chitosan hydrogel showed promising results in wound dressing, and bone fracture fixation devices^29^. The basic way to prepare a chitosan hydrogel is solubilization of chitosan in an acidic aqueous medium^30^.

PAA are used as ion exchange resins and adhesive polymers^31^. They are also known for being thickening, dispersing, suspending and emulsifying agents in pharmaceuticals, cosmetics and paints^32^. The neutralized PAA gels are appropriate to obtain biocompatible media for dermatological applications such as gels for skin care or treatment products for skin diseases^33, 34^.

The present study aims at overcoming the disadvantages of direct application of honey by formulation of honey-chitosan or honey-cabopol 934 hydrogels, to finally obtain a pharmaceutical honey topical preparation with desirable healing effects and antibacterial properties.

The present study measured the antibacterial activity of different honey formulations *in vitro* against *Pseudomonas aeruginosa*, *Staphylococcus aureus*, *Streptococcus pyogenes* and *Klebsiella pneumonia*. The most effective formulation was assessed for activity *in vivo*. Burn induced mice models were used as an assessment for wound healing and antibacterial effect of the honey – chitosan hydrogel *in vivo*.

## Materials and Methods

### Honey samples

Honey was purchased from local Emtinan Stores Giza, Egypt, carbopol 934 (Goodrich Chemicals Co., Cleveland, USA), chitosan low molecular weight was purchased from Sigma Chemical Company (Saint Louis, MO). Glacial acetic acid, triethanolamine (TEA), methyl paraben were purchased from Fisher Scientific, U.K. Analytical grade chemicals were used.

Six honey formulations were tested; 25%, 50% and 75% w/w; each concentration was formulated as a hydrogel twice; once with carbopol 934 and the other with chitosan.

### Honey hydrogel formulation

Six formulae of Honey hydrogel were prepared by cold mechanical method^19^. Polyacrylic acid polymer (carbopol 934) and chitosan hydrogel were prepared by dissolving the calculated amount of polymer in purified water with continuous stirring using a magnetic stirrer for 1 h till the polymer soaked in water. TEA was added with continuous stirring to neutralize the carbopol hydrogel to maintain the pH of the hydrogel. Followed by addition of the necessary amount of methyl paraben as a preservative and then left for 24 h for complete swelling and equilibration of polymer. Finally honey with different concentrations were added to the previous mixture with uninterrupted stirring till honey totally dispersed in the hydrogel. The final weight was completed to 100 g with the aqueous solution. The final formulations were packed in wide mouth glass containers covered with screw plastic lid and placed in refrigerator to complete the formation of hydrogel^20^.

### Physicochemical Evaluation of Honey hydrogel Formulae

#### Visual examination

The prepared honey formulations were examined for their color, consistency, homogeneity^35^ and existence of lumps by visual check after they were set in the container.

#### pH determination

One g of each hydrogel formulae was weighed and mixed with 25 ml of purified water. The pH of the mixture was measured using pH meter (Orion Research, Inc., USA), which was calibrated prior to each use with buffer solution 4.0, 7.0 and 9.0. Experiments were carried out in triplicates and average values were calculated^36^.

#### Swelling index

The swelling capacity is an important characteristic of wound healing formulations especially in exudating wounds. Due to their high fluid holding capacity they can absorb a moderate amount of the wound exudates by swelling which leads to formation of a dry bed of wound which further aids into healing process. To determine the swelling index of the prepared topical hydrogel, one gram sample from each formula was soaked into 5 ml phosphate buffer pH 5.5 and left for a specific time, and then the excess buffer was removed and the samples were weighted again. This test was done at time intervals after one and three hours. The following formula will be used to calculate the swelling ratio^37^.

$${\rm{Swelling}}\,{\rm{ratio}}=({\rm{Ws}}-{{\rm{W}}}_{0}/{{\rm{W}}}_{0})\times 100$$

where Ws is the weight of the swollen hydrogel at time t and W_0_ is the initial weight.

#### Spreadability measurement

The spreadability of the hydrogel formulation was determined, by pressing 0.5 g hydrogel between two horizontal plates (20 × 20 cm), then 5 g standardized weight was put on the upper plate and left for about 5 minutes where no more spreading was expected^38^. Diameters of spread circles were measured in cm and were taken as comparative values for spreadability. The results obtained were the average of three determinations^39^.

### *In vitro* release studies

Honey release from prepared hydrogels was studied using a dialysis bag technique^40^. Dialysis bags (Spectra/PorR Dialysis Membrane, MWCO:3.500, Spectrum Laboratories Inc., USA) were soaked before use in distilled water at room temperature for 12 h to remove the preservative, followed by rinsing thoroughly with distilled water. The dialysis bags were fitted on the dissolution tester apparatus I by attaching a stainless steel part to allow fixing the dialysis bags. A specific weight of honey hydrogel was placed in a measured volume (100 ml) of 5.5 pH phosphate buffer at 37 ± 0.5 °C and the quantity of released honey was measured at different time intervals by taking the absorbance of test sample using UV-Vis spectrophotometer (Shimadzu 1800, Japan). The absorbance of the released honey was measured at λ max 340 nm. All experiments were done in triplicates, the average of results were taken. Blank experiments were done using plain bases.

### *In vitro* antimicrobial examination of honey formulations

Disc Diffusion antibiotic sensitivity test (Kirby-Bauer) technique was employed as previously described^41^. The tests were done against the most strains known to be isolated from burn infections. The tests were run in Mueller Hinton agar (Oxoid, UK) for *Pseudomonas aeruginosa*, *Staphylococcus aureus* and *Klebsiella pneumonia*; Blood agar for *Streptococcus pyogenes*. All were prepared according to the manufacturer’s instructions. Cheesbrough^42^ method was used for preparing filter paper disks (6 mm diameter). Disks saturated with the six different honey formulations were employed in the study. The test was done a second time using discs impregnated with F3 (honey 75% - chitosan formula), pure honey and pure chitosan. The method of Koneman *et al*.^43^ was used to prepare 5 mL 0.5 McFarland standard in a sterile test tube. From the corresponding bacterial suspension an inoculum of each bacterial isolate was prepared. Briefly, 5–6 bacterial isolate colonies were emulsified in sterile distilled water and the turbidity was adjusted to 1.5 × 10^8^ CFU/mL (equivalent to 0.5 McFarland standard). This was followed by dipping a sterile cotton swab into the standardized bacterial suspension and inoculated on the corresponding agar plates evenly. Then plates were left to dry for 5 minutes. This was followed by placement of all formulation impregnated disks on the agar plates and forced lightly to confirm thorough contact with agar. A space of 20 mm was kept from the plates‘ edges to avoid intersection of inhibition zones. Amoxicillin disk (15 g) was used as a positive control. After 15 minutes all plates were incubated at 37 °C for 3–5 days. The plates were inspected and the diameter of the zone of inhibition was measured.

### *In vivo* burn healing evaluation

#### Animals and Experimental Design

A total of 10 albino mice (weight 30–35 g) were used. The animals were almost 8 weeks old. Animals were separately kept in clean polyethylene cages under usual experimental conditions of temperature 23 ± 2 °C. Mice were divided into 2 equal groups; males and females then anaesthetized with anaesthetic ether and shaved on the back with electric clipper then with a shaving cream. The shaved area was disinfected with 70% (v/v) ethanol. The burn wounds were induced as stated in literature^44^. Briefly, a cylindrical metal rod (10 mm diameter) was heated over an open flame for 30 seconds and pressed to the shaved and disinfected dorsal mouse skin surface for 20 seconds under light ether anesthesia. Four burning positions were induced on the back of each mouse; the four positions were treated with the following: F (Honey 75% - chitosan formula), H (Pure honey 100%), P (Positive control, standard wound healing cream, silver sulphadiazine) and N (Negative control, normal saline). Using a sterile cotton swab, all treatments (100 mg) were applied topically on each mouse once daily in a rotational manner (FHPN, HPNF, PNFH,……). Treatments were continued daily for nine days.

The mice were fed on commercial pellet and water *ad libitum* throughout the study. The experimental protocol was approved by the Animal Care and Use Committee of The British University in Egypt and the study was done according to the animal welfare guidelines and regulations.

The assessed wound healing parameters were the measurement of burn edge contraction, continuous monitoring of possible bacterial contamination and histopathological findings of the burned skin autopsy taken after nine days of treatment, in addition to the continuous assessment of any wound bacterial infection.

#### Measurement of wound area

The changes in burn edge diameter was measured (mm) using a digital caliper every day before treatments application. Burn edge contraction was expressed as the decrease of the original burn diameter. All measurements were recorded for further analysis.

### Microbial infection assessment of the induced burns

Surface swabs were collected from burn wounds before the daily application of the topical treatments. The total burned area was swabbed using a sterile cotton swab. Swab samples were taken from the burned wound area where the degree of burn is highest. Samples were homogenized in 4 mL sterile saline. Samples were immediately cultured on blood agar and MacConkey agar plates. MacConkey agar was used for the isolation of Gram negative bacteria while the blood agar was used for isolation and identification of Gram positive bacteria. After inoculation, plates were incubated at 37 °C for 24–48 h.

### Histopathological analysis

Autopsy samples were taken from the skin of mice in both groups; males and females. 4 samples from each mouse representing the positions of application, with a total of 40 samples; 20 males and 20 females, representing 10 of each application. The samples were fixed in 10% formalin solution for 24 h. Tap water was used to wash the samples then serial dilutions of alcohol (methyl, ethyl and absolute ethyl) were used to dehydrate the samples. Xylene was used to clear the specimens then embedded in paraffin at 56 °C in hot air oven for 24 h. Tissue blocks in paraffin bees wax were prepared for sectioning at 4 microns thickness using a sledge microtome. Prepared tissue sections were collected on glass slides, de-paraffinized and stained by hematoxylin & eosin stain (H&E) for examination through the light electric microscope^45^. Digital photomicrographs were captured at representative locations and the burns were evaluated for the extent of acanthosis, new blood capillaries formation and regeneration.

### Statistical analysis

The results are expressed as mean of triplicate measurements of inhibition zones of *in vitro* antimicrobial activity. The results of the *in vivo* wound diameter are expressed as means of 10 measurements groups daily for the 9 days ± SD. In the latter case Two way ANOVA was used.

Day 9 measurements were statistically analyzed using one way ANOVA test followed by Tukey post-hoc test. The difference was considered significant at p < 0.05.

### Data availability

The data generated and/or analysed during the current study are available from the corresponding author on request.

## Results and Discussion

### Preparation and Physicochemical Evaluation of Honey hydrogel Formulae

Hydrogels are high-water content materials prepared from cross-linked polymers that are able to provide sustained, local delivery of a variety of therapeutic agents^46–49^. The development of hydrogels from a variety of synthetic materials like carbopol 934 has been widely explored for the controlled delivery of many therapeutics with diversity in mechanical strengths and biological responses^50–53^. Additionally, polysaccharides as category of natural polymers have been interestingly used as the structural material in hydrogels. This is due to polymers biocompatibility, low toxicity, low immunogenicity and susceptibility to enzymatic degradation^54^. Of these, hydrogels using the natural polymer, chitosan, have received a great deal of attention due to their well-documented biocompatibility, low toxicity^55, 56^, and degradability by human enzymes^57^.

In the current study different honey hydrogel formulations were prepared using cold mechanical method utilizing two gel forming polymers either synthetic or natural naming carbopol 934 and chitosan, respectively to compare the effect of polymer type regarding the physicochemical characteristics of prepared hydrogel (Table 1).

**Table 1:** Composition of Honey hydrogel formulae (%w/w).

Formula No.	Honey	Carbopol 934	Chitosan	Methyl paraben	TEA*	GAA**	Purified water up to
F1	25	—	3.5	0.1	—	1	100
F2	50	—	3.5	0.1	—	1	100
F3	75	—	3.5	0.1	—	1	100
F4	25	1	—	0.1	q.s	—	100
F5	50	1	—	0.1	q.s	—	100
F6	75	1	—	0.1	q.s	—	100

*TEA: Triethanolamine, **GAA: Glecial acetic acid.

All formulations prepared represented homogenous, transparent three-dimensional networks which is considered as a good point to assist the monitoring process of wound healing progression. Water-soluble linear polymers of both natural and synthetic origin are either physically associated or chemically cross-linked to form the hydrogel. However, simple mixing the components of gel under the appropriate conditions usually produce polymer-based physical gel^31^. This process has an advantage over other crosslinking methods since it can be performed at room temperature and in physiological pH without using toxic and hard to remove crosslinking agents^58^. Therefore, it is always safe for clinical applications. Polysaccharides such as chitosan are reported in literature for the preparation of physically crosslinked hydrogels by hydrophobic modification^59^. Because the network formation by this interaction is purely physical, gel formation can be reversed^60^.

This is important for the biodegradation and drug release kinetics of therapeutics from hydrogels.

The result of drug content varies as shown in Table 2. The results indicated that the process employed to prepare hydrogel formulations in this study was capable of producing formulations with uniform drug content and minimal variability. Honey was dispersed uniformly throughout the hydrogel. The pH value of each honey-hydrogel is shown in Table 2. The pH of formulated hydrogels was found to be in the range of 4.3 to 6.8, which lies in the normal pH range of the skin^61^, which indicates the suitability of the formulations for application on the skin to avoid any irritation upon application. Hydrogel containing 75% honey was slightly acidic (pH 4.3, 4.7) compared to other 25, 50% honey hydrogels. This could be due to the acidic property of honey itself; pH being between 3.2 and 4.5^18^. Uniform application of paste to the skin is dependent on the spreadability parameter, where a good paste takes less time to spread and will have high credibility and spreadability^62^. Spreadability is important in patient compliance and helps in uniform application of gel to the skin. Spreadability of ideal formulation was found to be 8.6 ± 0.03 which was met by F3 containing 75% honey with chitosan gelling agent as shown in Table 2.

**Table 2:** Physiochemical properties of prepared honey hydrogels.

Formulae	General appearances	Homogeneity	Honey content %	pH*	Spreadability* cm
F1	Transparent Golden color	Homogenous	99.49 ± 0.85	5.1 ± 0.01	6.1 ± 0.02
F2			99.00 ± 0.31	4.9 ± 0.03	7.1 ± 0.04
F3			97.00 ± 0.54	4.34 ± 0.02	8.6 ± 0.03
F4			98.62 ± 0.80	6.8 ± 0.02	5.75 ± 0. 01
F5			98.76 ± 0.46	5.9 ± 0.03	6.08 ± 0.01
F6			97.27 ± 0.31	4.7 ± 0.03	7.75 ± 0.04

Swelling index was performed for all formulations (F1-F6) initially after 1 h and up to 3 hrs (Table 3). It was noticed that although all formulations showed rapid swelling due to the porous nature of the hydrogels offering large surface area allowing rapid uptake of the solvent but it was found that swelling index was inversely proportional to the concentration of honey. As the honey concentration increases there is decrease in swelling percentage. This is may be attributed to the viscosity of the polymer used which had major influence on swelling process. Results reflected also relation between swelling and type of the polymer, degree of cross-linking, ionic strength and presence of water^63^. Increase in cross–linker concentration shows a direct negative effect on hydrogel swelling index due to formation of tighter structure^64^. This is in agreement with our results as the lowest swelling index values ranged from 15% to 33%w/w belonged to hydrogels based on high cross-linker polymer carbopol 934.

**Table 3:** Swelling index of the prepared formulations.

Formula no.	Swelling ratio w/w after 1 h	Swelling ratio w/w after 3 h
F1	83% ± 0.18%	125% ± 0.50%
F2	65% ± 0.12%	105% ± 0.3%
F3	54% ± 0.15%	73% ± 1.12%
F4	33% ± 0.30%	40% ± 0.18%
F5	23% ± 0.45%	26% ± 1.2%
F6	15% ± 0.25%	20% ± 1.14%

The values are means of 3 replicates ± Standard deviation.

### *In vitro* release studies

*In vitro* release study showed that the release of honey from the hydrogel varied according to the type of gelling agent used and percentage of honey in the hydrogel. The results showed a concentration-dependent increase in the amount of honey diffused through cellophane bag of all hydrogel formulations (*i.e*., from 25% to 75%). It was also observed that among the prepared hydrogels the release of honey increased with increasing its concentration regardless the polymeric hydrogel used in the formulation. This may be attributed to the viscosity of the gel matrix in the hydrogel which is a major issue to address in evaluation of drug penetration from gels through the skin or artificial film and is used to measure the extrudability of a gel^18^. The decreasing viscosity of the hydrogels with increasing honey concentrations (from F1 to F3 and from F4 to F6) not only indicates that these hydrogel formulations would be easily extruded from their containers but also shows potential enhancement in the diffusivity of honey within the hydrogel network which consequently would ease flux^65^.

Also it is worth saying that the method by which the therapeutics is loaded into the hydrogels has direct impact on drug release behavior^66^. The hydrogels prepared by direct addition method as adopted in this study represents a typical release profile with rapid burst release of the drugs (up to 70%) during initial hydrogel swelling after transfer to *in vitro* media, followed by the extended release of the remaining drugs loaded within the gel network. However, highly cross-linked polymer showed lower burst release of drugs (10–25%)^67, 68^.

All formulations based on chitosan gelling agent showed an initial burst effect. This may be attributed to honey diffusion due to rapid gel swelling as previously mentioned and release of adsorbed honey in the direction of the surface of the gel matrix^69, 70^.

On the other hand, hydrogel formulae based on carbopol 934 produced the least percent of honey released from the hydrogels. This is justified since carbopol 934, like other high molecular weight polymers, demonstrates multiple cross-linkages per polymer producing more robust hydrogels with rheology similar to mayonnaise^71, 72^. Among all hydrogel formulations, F4 carbopol 934 based hydrogel showed superior sustained drug release. Carbopol 934 is a hydrophilic polyacrylic acid polymer and its carboxyl groups become highly ionized after neutralization with TEA, forming a gel due to electrostatic repulsion among charged polymer chains. Increasing pH of the prepared hydrogel to be suitable for skin application resulted in uncoiling of the polymer chains due to ionization of its carboxyl groups and subsequently forming a rigid gel^73^.

Hydrogels are a common form of topical application. They control the release of loaded therapeutics by diffusion through the hydrogel microporous structure into the skin^74–76^. Kinetic study of the *in vitro* results of all prepared honey hydrogels revealed that the hydrogels acquire diffusion controlled-release kinetics. In other words, the release of honey from prepared hydrogels is diffusion rate limited. As the release exponent ‘n’ was found to be ≤ 0.5 regarding all preparations which indicates Fickian diffusion mechanism.

**Figure 1:**
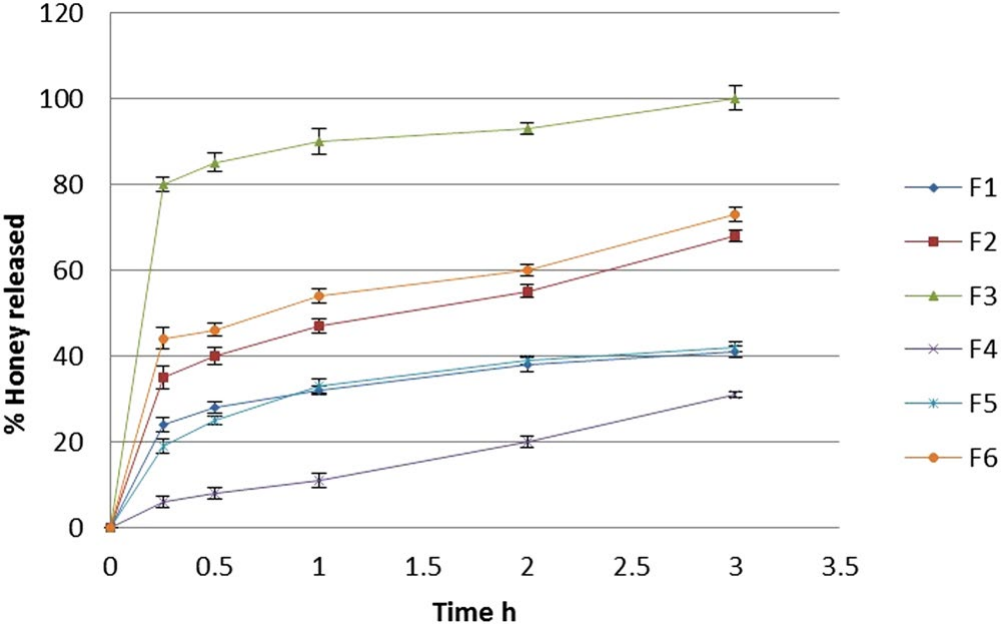
Honey release profile from prepared hydrogel formulae.

From the results (Fig. 1) it is seen that F3 formulation showed better *in vitro* honey release when compared with other formulations.

### *In vitro* antimicrobial examination of honey formulations

The antibacterial activity of the six prepared formulations (F1, F2, F3, F4, F5 and F6) was tested against the most common burn bacterial infections; *Pseudomonas aeruginosa*, *Staphylococcus aureus*, *Klebsiella pneumonia* and *Streptococcus pyogenes* (Table 4). The test was done using Disc Diffusion antibiotic sensitivity technique. All formulas prepared with chitosan showed larger zones of inhibition than those prepared with carbopol, thus better antibacterial activity. Among the three best chitosan formulations the diameter of zone of inhibition decreased with decreasing honey concentration. As the concentration of honey increased, the mean diameter of zone of inhibition increased till reaching maximum with honey 75% - chitosan gel formula where F3 formula showed the highest zone of inhibition compared to the others. Therefore, F3 formula was chosen for further investigations.

**Table 4:** Antibacterial activity of the six formulations (Honey-chitosan formula; F1, F2, F3 and honey-carbopol formula; F4, F5 and F6) against burn infection bacterial strains, determined by Disc Diffusion antibiotic sensitivity test.

Formula	Inhibition zone (mm) ± SD			
	*P. aeruginosa*	*S. aureus*	*K. pneumonia*	*S. pyogenes*
F1	8.3 ± 0.5	9.2 ± 0.4	8.7 ± 0.6	8.7 ± 0.6
F2	12.3 ± 0.8	13.9 ± 0.7	13.3 ± 0.8	13.6 ± 0.6
F3	21.5 ± 0.7	20.2 ± 0.4	19.5 ± 0.5	18.7 ± 0.4
F4	5.5 ± 0.4	5.9 ± 0.4	5.6 ± 0.4	5.7 ± 0.7
F5	6.0 ± 0.9	6.4 ± 0.6	6.2 ± 0.7	6.4 ± 0.8
F6	6.1 ± 0.5	6.8 ± 0.8	6.7 ± 0.6	6.5 ± 0.8

The values are means of 3 replicates ± Standard deviation.

**Figure 2:**
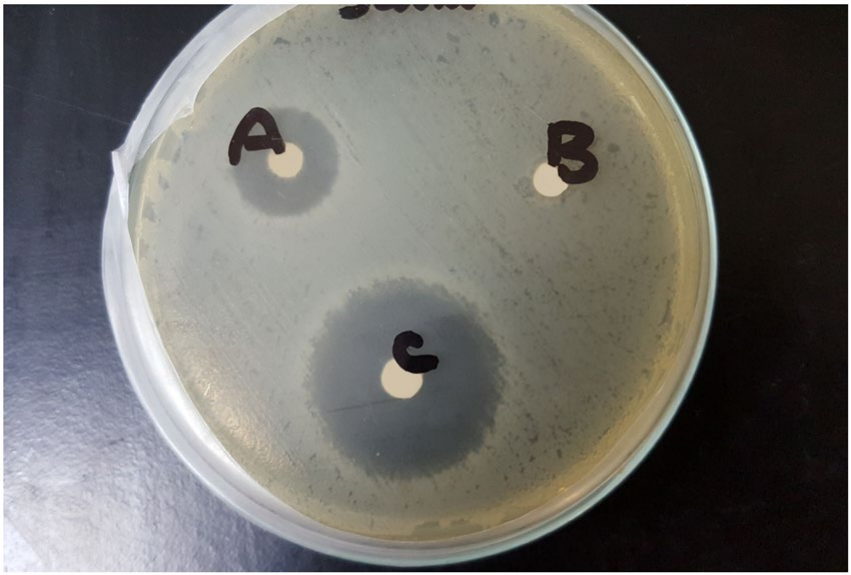
Disc Diffusion antibiotic sensitivity test against *Staphylococcus aureus*. (**a**) Pure honey, (**b**) Chitosan hydrogel, (**c**) F3 (75% honey + chitosan hydrogel).

A second Disc Diffusion antibiotic sensitivity test was done to compare the antibacterial activity of the chosen formula F3 with that of blank chitosan and pure honey (Table 5). Blank chitosan gel and pure honey showed minimum bacterial inhibition compared to F3 (Fig. 2). This can be explained by the known antimicrobial property of chitosan and the hygroscopic property of honey which dehydrate bacteria, also its high sugar content and low level pH can also prevent the microbes from growth^77^. It was also reported in literature that the 75% honey solution exerted higher antibacterial activity than pure honey against common respiratory tract infections^5^.

**Table 5:** Antibacterial activity of honey 75% - chitosan formula (F3), blank chitosan and pure honey against burn infection bacterial strains, determined by Disc Diffusion antibiotic sensitivity test.

Formula	Inhibition zone (mm) ± SD			
	*P. aeruginosa*	*S. aureus*	*K. pneumonia*	*S. pyogenes*
F3	21.5 ± 0.7	20.2 ± 0.4	19.5 ± 0.5	18.7 ± 0.4
Blank chitosan	7.2 ± 0.5	7.8 ± 0.6	8.1 ± 0.7	8.0 ± 0.7
Pure honey	13.8 ± 1	15.1 ± 0.9	14.0 ± 0.8	14.2 ± 1.1

The values are means of 3 replicates ± Standard deviation.

Therefore, it is evident that both chitosan and honey contribute to the antibacterial property in the prepared gel and the addition of honey increases the zone of inhibition.

### *In vivo* burn healing evaluation

**Figure 3:**
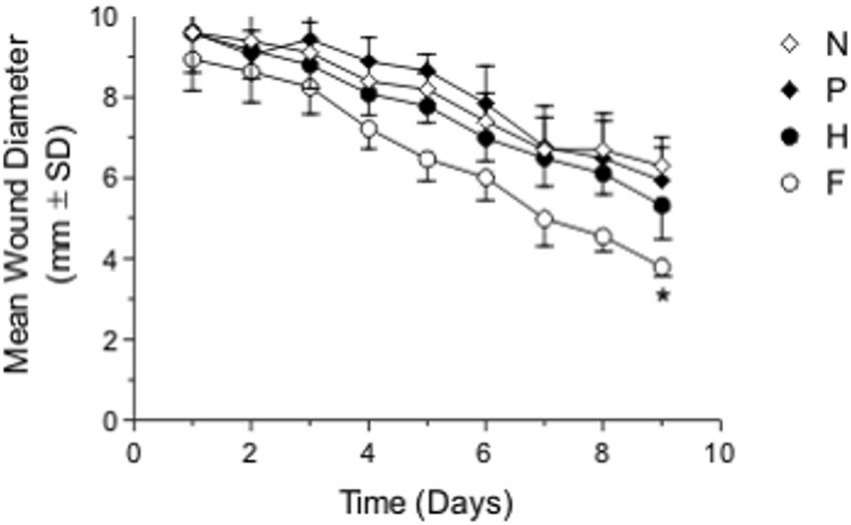
Mean wound diameter (mm ± SD). N: negative control, normal saline, P: positive control, silver sulphadiazine, H: pure honey, F3: 75% honey-chitosan formula. *F3 significantly effective than either control, P or H (p < 0.001).

Burns were induced in albino mice using a preheated metallic rod which gave a final diameter in all burned positions of about 10 mm (Fig. 3). Treatments were applied on daily basis for nine days.

### Measurement of wound area

Measurement of wound diameter was a main criteria for indication of progressive healing where burn edge contraction was expressed as a decrease of the original burn diameter. All measurements were recorded daily for nine days. The positions treated with F3 formula on all mice showed continuous burn edge contraction during the course of treatment. At the end of the treatment period the maximum burn diameter contraction was reached by F3. This shows that honey 75% - chitosan formula showed the best healing properties compared to the pure honey and the commercial product tested (Table 6).

**Table 6:** Wound diameter (mm) and standard deviation from Day of burn induction till end of treatment course.

Days	Wound diameter (mm)			
	F3	H	P	N
0	10	10	10	10
1	8.9 ± 0.8	9.6 ± 1	9.6 ± 1	9.6 ± 1.1
2	8.6 ± 0.8	9.2 ± 0.7	9.1 ± 0.6	9.4 ± 0.9
3	8.3 ± 0.7	8.8 ± 0.6	9.4 ± 0.4	9.1 ± 1.1
4	7.2 ± 0.5	8.1 ± 0.5	8.9 ± 0.6	8.4 ± 0.5
5	6.5 ± 0.6	7.8 ± 0.4	8.7 ± 0.4	8.2 ± 0.4
6	6.0 ± 0.6	7.0 ± 0.6	7.8 ± 0.9	7.4 ± 0.7
7	5.0 ± 0.7	6.5 ± 0.7	6.8 ± 1	6.7 ± 0.8
8	4.5 ± 0.4	6.1 ± 0.5	6.5 ± 0.9	6.7 ± 0.9
9	3.8 ± 0.2	5.3 ± 0.8	5.9 ± 0.8	6.3 ± 0.7

Wound diameter: average diameter of 10 respective position of treatment on mice daily.

F3 is significantly effective than either control, P or H.

F3: honey 75%-chitosan formula.

H: pure honey.

P: silver sulphadiazine commercial treatment (positive control).

N: Normal saline (negative control).

SD: standard deviation.

### Microbial infection assessment of the induced burns

Skin swabs were taken continuously from the surface of the burn to investigate any bacterial infection of the wound. All swabs from all positions did not show any bacterial growth during the course of treatment, which means that no bacterial infection occurred to the induced burns. This may be attributed to the already known antibacterial properties of the treatments (honey, chitosan and commercial product). It was reported that during treatment with honey the wounds become sterile within 7–10 days and promote the formation of healthy granulation tissue^78^. The slight acidic pH of the prepared formula (pH 4.3–4.7), similar to that of healthy skin, makes it most comfortable to wear and this low pH creates an unfavorable environment for bacterial growth^79^. In previous experimentally induced burns, there was no obvious infection, but honey continued to cause a decrease in inflammation, this shows that the anti-inflammatory activity of honey is a direct action and not a side effect of eliminating infection by antibacterial activity^80^.

Swabs from positions treated with normal saline (0.9% NaCl) also did not show bacterial growth this may be attributed to the fact that normal saline is isotonic and it does not induce tissue damage, cause sensitization or allergies, or alter the normal bacterial flora of the skin which makes it the favored wound cleansing solution for non-infected wounds because it does not interfere with the normal healing process^77^.

### Histopathological analysis

Forty autopsy samples were cut and divided into four groups according to the treatment applied. All skin samples were compared to a control group.

The skin autopsy of the control group showed no histopathological alteration where the skin showed the normal histological structure of the epidermis, dermis, subcutaneous tissue with adipose one and underlying musculature.

**Figure 4:**
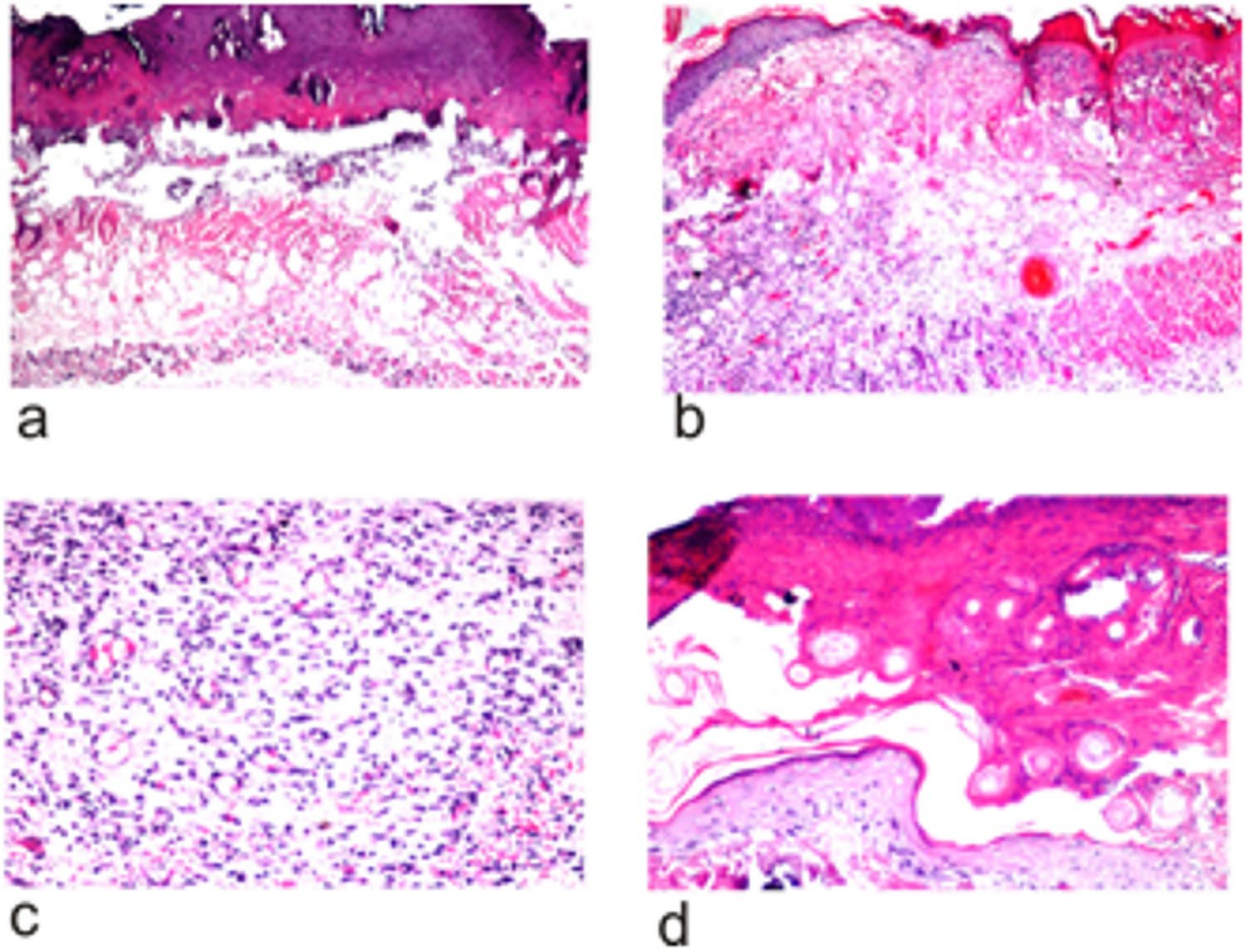
Representative photomicrographs of tissue sections stained with H&E. (**a**) Day 1 directly after induced third degree burn showing necrosis of the epidermal and dermal layers in focal manner with inflammatory cells infiltration in subcutaneous tissue and musculature (16x mag). (**b**) Day 9 after burn. Skin of mice treated with silver sulphadiazine showing focal necrosis of the epidermis with acanthosis in the adjacent one and inflammatory cells infiltration in the underlying dermis and subcutaneous tissue (16x mag). (**c**) Day 9 after burn. Skin of mice treated with pure honey showing few newly formed blood capillaries and few fibroblastic cell proliferation as granulation tissue in subcutaneous tissue with inflammatory cell infiltration (40x mag). (**d**) Day 9 after burn. Skin of mice treated with honey 75%-chitosan formula showing regeneration of the epidermal layer with separation of the necrosed areas as scales and formation of new blood capillaries. (40x mag).

The resulting burn was a third degree type which showed a focal wide area of necrosis in the epidermis, dermis and underlying subcutaneous tissue associated with massive inflammatory cells infiltration in the subcutaneous tissue as well as adipose tissue and musculature (Fig. 4a).

The epidermal layer of the group of thermally inducted wound of mice and treated by silver sulphadiazine showed focal necrosis with acanthosis in the adjacent one. Inflammatory cells infiltration was detected in the underlying dermis and the deep subcutaneous tissue as well as the adipose tissue. Inflammatory cells infiltration was also detected with necrosis and congestion in the blood vessels in the underlying musculature (Fig. 4b).

On the other hand, the group treated by 100% honey, there was rejection of the necrosed area from the epidermis (scales) associated with acanthosis in the prickle cell layer of the adjacent epidermis. The subcutaneous tissue showed inflammatory cells infiltration as well as few fibroblastic cells proliferation and few newly formed capillaries as beginning of granulation tissue formation. Inflammatory cells infiltration was detected in between the degenerated musculature (Fig. 4c).

Regarding the group treated by 75% honey-chitosan formula, the epidermis and dermis showed regeneration with hyperkeratosis and acanthosis in the epidermis replacing the necrosed one. These findings are consistent with previous studies which showed that honey promotes epithelization of wounds^81–85^ where epithelium regeneration is one of the most important indices for wound healing rate evaluation. Few inflammatory cells infiltration was detected in the dermis and subcutaneous tissue associated with fibroblastic cells proliferation and newly formed blood capillaries which is an indication of the beginning of healing process as stated by previous researches^86–91^ (Fig. 4d).

From the histopathological sections it is clear that treatment with honey 75%-chitosan (F3) treatment resulted in best healing results indicated by the regeneration of the epidermis tissue and the formation of new blood capillaries. This was followed by pure honey treatment (H) also showed a promising healing effect but to a lesser extent compared to the F3 formula. Finally the least effect was the commercial formulation where no epithelial regeneration or new blood capillaries were formed.

### Statistical analysis

There was a significant healing of wounds by time in all groups (*p* < *0.0001*), significant difference between groups (*p* < *0.0001*) using two way ANOVA, as well as a significant time x treatment interaction (*p* < *0.0018*) at day 9 of treatment.

## Conclusion

This study shows that honey plays a positive role in modulating wound healing when incorporated into chitosan-based hydrogel matrix. The prepared hydrogel wound dressing containing 75% of honey will not function merely as coverage to provide clean moist environment for healing but also directly contribute to enhanced tissue regeneration and recovery.

These design parameters established an alternative cheap, non-toxic, natural and efficient honey-chitosan hydrogel delivery system that can move closer to clinical availability for wound healing.
